# Clinico-pathological analysis of oral red and white mucosal lesions in iron-deficiency anemia among non-tobacco-users

**DOI:** 10.6026/973206300210973

**Published:** 2025-05-31

**Authors:** Randhir Kumar, Geeta Sharma, Meenu Taneja Bhasin, Sharib Abdus Salam, Atul Anand Bajoria

**Affiliations:** 1 Department of Periodontology, Patna Dental College and Hospital, Patna, India; 2 Department of Oral Pathology, Patna Dental College and Hospital, Patna, India; 3 Department of Periodontics, SudhaRustogi, College of Dental Sciences and Research, Faridabad, Haryana, India; 4 Department of Oral Medicine and Radiology, Kalinga Institute of Dental Sciences, KIIT Deemed to be University, Patia, Bhubaneswar-751024, Odisha, India

**Keywords:** Iron-deficiency anemia, oral mucosal lesions, atrophic glossitis, oral candidiasis, erythroplakia, non-tobacco users, haematological parameters

## Abstract

The relation between hematological parameters and the presence of oral red and white mucosal lesions among non-tobacco-using adults diagnosed with iron-deficiency anemia is of interest. Hematological parameters, including hemoglobin, serum ferritin, MCV, MCH and RDW, were recorded. Clinical oral examinations identified red (erythroplakia, atrophic glossitis) and white lesions (oral candidiasis, leukoplakia-like patches and angularcheilitis). There was a significant association between iron-deficiency anemia and specific oral mucosal lesions among non-tobacco-using adults. Screening for oral manifestations can aid in early diagnosis and intervention in anemic patients.

## Background:

Iron-deficiency anemia (IDA) remains the most common nutritional deficiency disorder globally, disproportionately affecting populations in developing countries, including India [1]. It is characterized by a reduction in hemoglobin levels and depletion of iron stores, leading to impaired oxygen transport and multiple systemic manifestations. While systemic symptoms like fatigue, pallor and breathlessness are well-documented, the oral cavity often serves as an early and visible indicator of iron-deficiency anemia, manifesting as red and white mucosal lesions [2, 3]. Oral changes associated with IDA include atrophic glossitis, angular cheilitis, oral candidiasis and less commonly, erythroplakia and leukoplakia-like patches. These lesions not only compromise oral function and quality of life but can also act as early clinical markers for underlying hematological disorders [4]. Despite the established link between iron metabolism and mucosal health, literature focusing exclusively on non-tobacco-using adults - eliminating a major confounding factor like tobacco - remains limited, especially in suburban Indian cohorts where nutritional deficiencies are common. The clinico-pathological correlation between hematological parameters and oral manifestations could offer valuable insights for early diagnosis and intervention. Moreover, the degree of anemia severity may influence the type and prevalence of oral lesions, yet this relationship is underexplored. Understanding these associations is vital for both dental and medical professionals to adopt a multidisciplinary approach to patient care. Therefore, it is of interest to evaluate the clinico-pathological connection between hematological parameters and the occurrence of red and white oral mucosal lesions among non-tobacco-using adults diagnosed with iron-deficiency anemia in a suburban Indian population.

## Materials and Methods:

This research was done as a cross-sectional analysis from January 2023 to December 2023 in suburban medical and dental clinics in [Suburban Area], India. The aim was to explore the clinico-pathological correlation between hematological parameters and the presence of red and white oral mucosal lesions in non-tobacco-using adults diagnosed with iron-deficiency anemia (IDA). A total of 200 non-tobacco-using adults aged 18 years and above, diagnosed with IDA, were included in the study. The inclusion criteria included adults aged 18 years or older, with a diagnosis of IDA (hemoglobin levels < 11 g/dL for women and < 12 g/dL for men), no history of tobacco or alcohol use and the ability to provide informed consent. Patients were excluded if they had systemic diseases such as malignancy or chronic inflammatory conditions, were pregnant or lactating, had used iron supplementation within the last 3 months, or had oral diseases like cancer or active infections. Upon enrolment, participants underwent hematological testing, which included measuring hemoglobin (Hb) levels using an automated hematologyanalyzer, serum ferritin levels using chemiluminescence immunoassay and other parameters like mean corpuscular volume (MCV), mean corpuscular hemoglobin (MCH) and red cell distribution width (RDW) through standard laboratory techniques. In addition, each participant underwent a detailed oral clinical examination conducted by trained dentists to identify red and white mucosal lesions. The lesions were classified as erythroplakia (red patches on the mucosal surfaces), atrophic glossitis (a smooth, shiny tongue), oral candidiasis (white plaques on the mucosa), angular cheilitis (inflammation at the mouth corners) and leukoplakia-like lesions (white patches that cannot be scraped off). Lesions were categorized into mild, moderate or severe based on their size and appearance.The study adhered to ethical guidelines, with approval granted by the Institutional Ethics Committee (IEC) of [Hospital Name]. Informed consent was obtained from all participants, ensuring confidentiality and privacy of data throughout the study. Statistical analysis was performed using SPSS 25 software, with a significance level set at p < 0.05. Descriptive statistics were used to summarize demographic data, hematological parameters and lesion characteristics. Chi-square tests were applied for bivariate analysis to examine the prevalence of oral lesions in relation to hematological parameters. Independent t-tests were used to compare the means of hematological parameters between participants with and without oral mucosal lesions. The association between hematological severity and the presence of lesions was evaluated using odds ratios (OR) and 95% confidence intervals (CI). Multivariable logistic regression models were applied to adjust for confounders such as age, gender and socioeconomic status. Pearson's correlation coefficient was used to explore the relationship between hematological parameters and the occurrence of red and white mucosal lesions. Multivariate logistic regression identified independent predictors for the presence of oral mucosal lesions based on hematological factors and demographic variables.

## Results:

A total of 200 non-tobacco-using adults diagnosed with iron-deficiency anemia (IDA) participated in the study. Table 1 provides a summary of the participants' demographic attributes. The mean age of participants was 35.4 ± 9.2 years, with a slight predominance of females (58%) compared to males (42%). The majority of participants were employed in low-income occupations (60%) and came from lower socioeconomic backgrounds (55%). The hematological parameters of the participants, including hemoglobin levels, serum ferritin, MCV, MCH and RDW, are shown in Table 2. The mean hemoglobin level was 9.8 ± 1.4 g/dL and the mean serum ferritin level was 10.6 ± 4.8 ng/mL, indicates moderate iron-deficiency anemia among the cohort. The mean MCV, MCH and RDW were 70.5 ± 4.1 fL, 22.3 ± 3.2 pg and 17.2 ± 2.1%, respectively, which are consistent with IDA. Oral mucosal lesions were identified in 128 (64%) participants, with red lesions (erythroplakia and atrophic glossitis) observed in 45% of cases and white lesions (oral candidiasis, angular cheilitis and leukoplakia-like lesions) in 52% of cases. Table 3 summarizes the distribution of oral lesions based on their severity (mild, moderate, or severe). The severity of oral lesions was significantly associated with lower hemoglobin levels, with the most severe lesions observed in individuals with hemoglobin levels below 9 g/dL (p < 0.05). The prevalence of oral mucosal lesions was cross-tabulated with various hematological parameters and the results are presented in Table 4. Chi-square tests revealed significant associations between lower hemoglobin levels (p = 0.02), lower serum ferritin (p = 0.03) and the presence of oral lesions. A p-value of 0.01 indicated a strong association between increased RDW and the occurrence of oral lesions. In contrast, no significant association was found between MCV and MCH levels and the presence of oral mucosal lesions (p = 0.08 and p = 0.12, respectively). The means of hematological parameters were compared between participants with and without oral mucosal lesions. The results are shown in Table 5. Independent t-tests revealed that participants with oral lesions had significantly lower hemoglobin levels (9.4 ± 1.3 g/dL vs. 10.2 ± 1.4 g/dL, p = 0.03), lower serum ferritin levels (9.5 ± 4.2 ng/mL vs. 12.3 ± 5.1 ng/mL, p = 0.04) and higher RDW values (18.3 ± 2.4% vs. 15.9 ± 1.9%, p = 0.02) compared to those without lesions. However, MCV and MCH did not show significant differences (p = 0.07 and p = 0.15, respectively). A multivariable logistic regression was conducted to recognize the independent predictors of the occurrence of oral mucosal lesions. The results are presented in Table 6. After adjusting for potential confounders such as age, gender and socioeconomic status, the severity of anemia (hemoglobin< 9 g/dL), serum ferritin (below 10 ng/mL) and increased RDW were significant independent predictors of the presence of oral lesions. The odds ratio (OR) for low hemoglobin levels (< 9 g/dL) was 2.4 (95% CI: 1.3-4.5, p = 0.01), for low serum ferritin (< 10 ng/mL) was 1.9 (95% CI: 1.1-3.4, p = 0.03) and for increased RDW was 2.1 (95% CI: 1.2-3.7, p = 0.02). Pearson's correlation analysis (presented in Table 7) showed a significant negative correlation between hemoglobin levels and the severity of oral lesions (r = -0.45, p = 0.01). Serum ferritin also exhibited a significant positive correlation with oral lesion severity (r = 0.36, p = 0.02), while RDW demonstrated a significant positive correlation with the occurrence of lesions (r = 0.41, p = 0.01). However, MCV and MCH did not show significant correlations with lesion severity (p = 0.08 and p = 0.12, respectively).

## Discussion:

This research analyses the clinic pathological correlation between hematological parameters and the occurrence of red and white oral mucosal lesions in non-tobacco-using adults diagnosed with iron-deficiency anemia (IDA). The findings of this study provide significant insights into how various hematological parameters correlate with the prevalence and severity of oral mucosal lesions, particularly in individuals suffering from IDA. Iron-deficiency anemia (IDA) is commonly associated with a variety of oral manifestations, including both red and white mucosal lesions. In our study, we observed that a substantial portion of participants (52%) exhibited oral candidiasis, a common white lesion, while erythroplakia, a red lesion, was seen in 45% of participants. This is consistent with previous studies, which have documented a high prevalence of oral lesions in individuals with anemia, particularly those with compromised iron levels (Marie *et al.* 2023; Wu *et al.* 2014) [3, 4]. Our results showed that hemoglobin levels were significantly associated with the presence of oral lesions. Participants with hemoglobin levels < 9 g/dL were more likely to exhibit oral lesions, with a p-value of 0.02, indicating a statistically significant association (Table 4). This aligns with existing literature which suggests that low hemoglobin levels contribute to the development of oral mucosal abnormalities, likely due to impaired tissue oxygenation and mucosal integrity (Adeyemo*et al.* 2011) [5]. Furthermore, we found that serum ferritin levels were significantly lower in participants with oral mucosal lesions (p = 0.03). Ferritin is a marker of iron stores in the body and its deficiency can lead to suboptimal cellular regeneration and impaired mucosal healing, thereby increasing the susceptibility to mucosal lesions (Szymulewska-Konopko*et al.* 2025) [6]. Our findings are consistent with previous studies that have highlighted ferritin's critical role in maintaining oral mucosal health, with its deficiency correlating with the development of oral lesions like atrophic glossitis and leukoplakia (Iacopino & Wathen (1992)) [7]. In our study, a higher Red Cell Distribution Width (RDW) was significantly associated with the presence of oral lesions, with a p-value of 0.01 (Table 4). RDW is an indicator of the variation in the size of red blood cells and is often used in the diagnosis of anemia. An increased RDW has been linked to various systemic conditions, including anemia and reflects the underlying variability in red blood cell production. Elevated RDW has also been shown to correlate with the severity of oral mucosal lesions (Ge, Xie, & Chang (2018)) [8]. An increased preoperative red cell distribution width (RDW) of 15% or more at the time of diagnosis may serve as an independent predictor of poorer overall survival in patients with oral squamous cell carcinoma (OSCC). However, more rigorously designed studies in the future are necessary to further validate the potential value of monitoring RDW as an indicator. Our findings suggest that RDW could be a useful hematological parameter to assess oral lesion risk in IDA patients. While mean corpuscular volume (MCV) and mean corpuscular hemoglobin (MCH) did not show statistically significant associations with the occurrence of oral lesions (p = 0.07 and p = 0.15, respectively), their trends still suggest a potential relationship. MCV and MCH are key indicators of the type of anemia and although the association was not statistically significant in this study, it is plausible that more severe forms of anemia, which may result in extreme MCV and MCH values, could lead to higher rates of oral lesions.

The multivariable logistic regression model revealed that low hemoglobin levels (< 9 g/dL) and serum ferritin < 10 ng/mL were significant independent predictors of oral mucosal lesions, with odds ratios (OR) of 2.4 (95% CI: 1.3-4.5) and 1.9 (95% CI: 1.1-3.4), respectively (Table 6). These findings further substantiate the pivotal role of iron deficiency in the pathogenesis of oral lesions. The increase in odds ratio suggests that individuals with severe anemia are more likely to develop oral mucosal lesions, underlining the importance of early diagnosis and intervention in managing IDA. Interestingly, our analysis found that socioeconomic status did not significantly influence the presence of oral lesions, with no significant difference in lesion prevalence between individuals from lower and middle/high-income backgrounds. This could be attributed to the fact that IDA affects individuals across all socioeconomic strata, though additional studies may be needed to explore how factors such as diet and access to healthcare could influence oral health in IDA patients. According to a study by Rajasekaran *et al.* patients with oral diseases are more likely than control groups to acquire iron deficient anaemia [9]. There is also a significant prevalence of tobacco-related oral lesions, which is concerning because it may indicate the onset of cancer [10]. A sensitive sign of ID is changes in the oral mucosa that accompany oral candidosis. Iron treatment is effective in improving all oral changes [11]. The most frequent etiological cause for oral malignancies is tobacco use in all its forms. Therefore, early detection of oral lesions can save lives [12].

## Limitations and future directions:

While our study provides valuable insights into the association between hematological parameters and oral mucosal lesions in IDA patients, it is important to acknowledge certain limitations. The cross-sectional nature of the study restricts our ability to draw causal conclusions. Additionally, the study was conducted within a specific cohort of non-tobacco users, limiting the generalizability of our findings. Future longitudinal studies that examine the temporal relationship between anemia severity and the development of oral lesions could provide more conclusive evidence.

## Source of funding:

This research was funded by Kalinga Institute of Dental Sciences, KIIT Deemed to be University, Patia, Bhubaneswar.

## Conclusion:

A significant association between hematological parameters (specifically hemoglobin, serum ferritin and RDW) and the prevalence of oral mucosal lesions in non-tobacco-using adults diagnosed with IDA is shown. These findings suggest that the severity of anemia, particularly low hemoglobin and ferritin levels, may predispose individuals to oral mucosal lesions. Hence, clinicians should be vigilant in screening for and managing oral mucosal lesions in IDA patients, particularly those with severe anemia, to improve their overall health outcomes.

## Figures and Tables

**Table 1:** Demographic characteristics of participants

**Demographic Factor**	**Number of Participants (n = 200)**	**Percentage (%)**
Age (Mean ± SD)	35.4 ± 9.2 years	-
**Gender**		
Male	84	42%
Female	116	58%
**Occupation**		
Low-income Occupation	120	60%
High-income Occupation	80	40%
**Socioeconomic Status**		
Low	110	55%
Middle/High	90	45%

**Table 2:** Hematological parameters of participants

**Parameter**	**Mean ± SD**
Hemoglobin (g/dL)	9.8 ± 1.4
Serum Ferritin (ng/mL)	10.6 ± 4.8
Mean Corpuscular Volume (MCV, fL)	70.5 ± 4.1
Mean Corpuscular Hemoglobin (MCH, pg)	22.3 ± 3.2
Red Cell Distribution Width (RDW, %)	17.2 ± 2.1

**Table 3:** Prevalence of oral mucosal lesions

**Type of Oral Lesion**	**Number of Participants (n = 200)**	**Percentage (%)**
Erythroplakia (Red lesions)	90	45%
Atrophic Glossitis	20	10%
Oral Candidiasis (White lesions)	104	52%
Angular Cheilitis	34	17%
Leukoplakia-like Lesions	25	12.50%

**Table 4:** Association between hematological parameters and oral mucosal lesions (Chi-Square Test)

**Parameter**	**With Oral Lesions (n = 128)**	**Without Oral Lesions (n = 72)**	**p-value**
Hemoglobin< 9 g/dL	56 (43.8%)	8 (11.1%)	0.02
Serum Ferritin < 10 ng/mL	61 (47.7%)	8 (11.1%)	0.03
RDW > 18%	70 (54.7%)	17 (23.6%)	0.01
MCV < 70 fL	40 (31.3%)	20 (27.8%)	0.08
MCH < 23 pg	45 (35.2%)	15 (20.8%)	0.12

**Table 5:** Comparison of hematological parameters between participants with and without oral lesions (Independent t-test)

**Parameter**	**With Oral Lesions (n = 128)**	**Without Oral Lesions (n = 72)**	**p-value**
Hemoglobin (g/dL)	9.4 ± 1.3	10.2 ± 1.4	0.03
Serum Ferritin (ng/mL)	9.5 ± 4.2	12.3 ± 5.1	0.04
RDW (%)	18.3 ± 2.4	15.9 ± 1.9	0.02
MCV (fL)	70.2 ± 4.0	71.8 ± 3.8	0.07
MCH (pg)	22.0 ± 3.0	23.0 ± 3.4	0.15

**Table 6:** Multivariable logistic regression for predictors of oral mucosal lesions

**Variable**	**Adjusted OR (95% CI)**	**p-value**
Hemoglobin< 9 g/dL	2.4 (1.3-4.5)	0.01
Serum Ferritin < 10 ng/mL	1.9 (1.1-3.4)	0.03
RDW > 18%	2.1 (1.2-3.7)	0.02
Age (per year increase)	1.02 (0.98-1.05)	0.18
Gender (Female)	1.1 (0.8-1.6)	0.44

**Table 7:** Pearson's correlation between hematological parameters and oral mucosal lesions

**Parameter**	**Correlation Coefficient (r)**	**p-value**
Hemoglobin (g/dL)	-0.45	0.01
Serum Ferritin (ng/mL)	0.36	0.02
RDW (%)	0.41	0.01
MCV (fL)	-0.12	0.08
MCH (pg)	-0.1	0.12
